# Neural mechanisms underlying sensitivity to reverse-phi motion in the fly

**DOI:** 10.1371/journal.pone.0189019

**Published:** 2017-12-20

**Authors:** Aljoscha Leonhardt, Matthias Meier, Etienne Serbe, Hubert Eichner, Alexander Borst

**Affiliations:** 1 Max-Planck-Institute for Neurobiology, Martinsried, Germany; 2 Graduate School for Systemic Neurosciences, Munich, Germany; University of California, Los Angeles, UNITED STATES

## Abstract

Optical illusions provide powerful tools for mapping the algorithms and circuits that underlie visual processing, revealing structure through atypical function. Of particular note in the study of motion detection has been the reverse-phi illusion. When contrast reversals accompany discrete movement, detected direction tends to invert. This occurs across a wide range of organisms, spanning humans and invertebrates. Here, we map an algorithmic account of the phenomenon onto neural circuitry in the fruit fly *Drosophila melanogaster*. Through targeted silencing experiments in tethered walking flies as well as electrophysiology and calcium imaging, we demonstrate that ON- or OFF-selective local motion detector cells T4 and T5 are sensitive to certain interactions between ON and OFF. A biologically plausible detector model accounts for subtle features of this particular form of illusory motion reversal, like the re-inversion of turning responses occurring at extreme stimulus velocities. In light of comparable circuit architecture in the mammalian retina, we suggest that similar mechanisms may apply even to human psychophysics.

## Introduction

In complex environments, visual motion represents a cue of critical ecological importance. Organisms from humans to insects derive information about movement from optical signals, putting it to use during course control, spatial navigation, or object tracking [1]. Remarkably, visual systems appear to converge on similar solutions: critical operations in elementary motion detection, like spatially asymmetric temporal filtering followed by non-linear interaction, as well as general architecture, like the separate processing of positive (ON) and negative (OFF) contrast, are preserved between for instance mouse and fly [2].

Optical illusions lend particular weight to these findings by showing that even non-ecological functions of a given algorithm can generalize across organisms, which indicates substantial overlap in mechanism. A particularly prominent example is the reverse-phi effect. When one combines discretized pattern motion, closely related to so-called phi motion defined by temporally successive, spatially adjacent flashes [3], with reversals of visual contrast, the detected direction is generally opposite to that of the contrast-preserving pattern. This applies to simple visual reflexes and neural responses [4–9] as well as to conscious perception by human observers [10] and over a wide range of stimulus types. Phenomenological and biophysical models of motion detection account for the effect by means of various mechanisms, ranging from feature correspondence at higher processing levels [3] to algorithms based on detection of motion energy or, equivalently, cross-correlation [4,5,11–20] and estimation of spatiotemporal gradients [21–24]. However, a circuit-level understanding of the illusion remains elusive.

**Fig 1:**
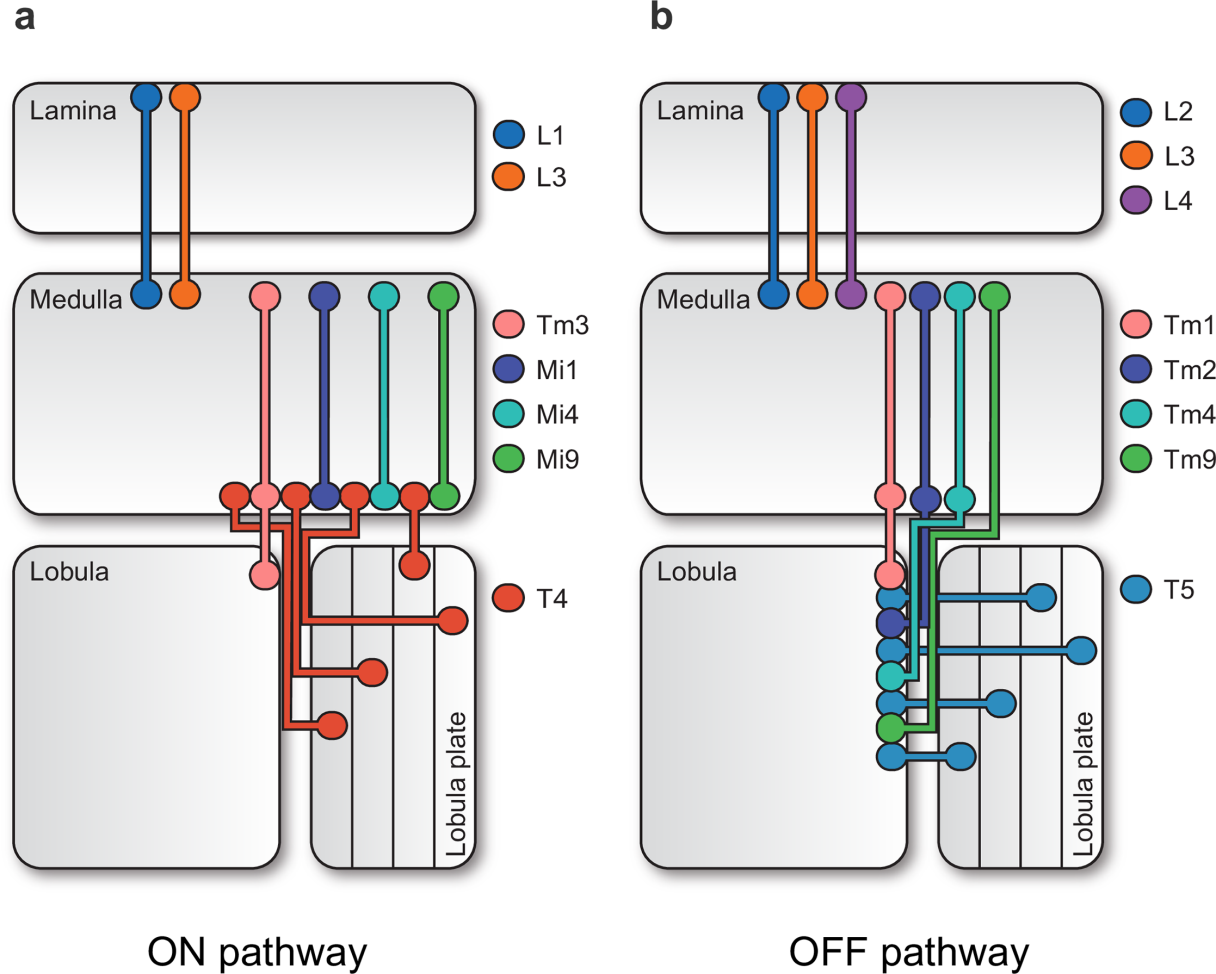
The neural architecture of motion detection in the fruit fly. **a** Stylized rendering of neuropils and cells involved in generating local and global motion signals in the ON-selective T4 pathway. **b** Corresponding schematic for the OFF-selective T5 pathway. Briefly, optical signals are transduced by photoreceptors in the retina, split into ON and OFF signals at the lamina level, processed by an array of interneurons in the medulla, and become direction selective through interactions on T4 and T5 dendrites. Finally, lobula plate tangential cells (LPTCs) select and pool appropriate local signals from four directionally tuned layers to generate complex flow field sensitivities.

In recent years, *Drosophila melanogaster* emerged as a particularly powerful model organism for the study of motion detection. Genetic access through binary expression systems like Gal4-UAS [25,26] and a robust repertoire of motion-driven behaviors, such as the optomotor reflex [1], have combined to paint an ever clearer picture of how neural circuits accomplish direction selectivity. Briefly, photons are transduced in the ommatidia of the fly retina. The resulting signals are spatially and temporally processed in neuropils called lamina and medulla and then non-linearly combined on dendrites of motion-sensitive cell types T4 and T5 in medulla and lobula, respectively, to generate local responses specific to one direction in visual space. Critically, this process occurs twice: once for motion defined by positive luminance changes (ON motion; from cells L1 and L3 onto T4 via Mi1, Tm3, Mi4, and Mi9; **Fig 1A**) and once for motion defined by negative luminance changes (OFF motion; from cells L2-4 onto T5 via Tm1, Tm2, Tm4, Tm9; **Fig 1B**) [5,27–38]. Wide-field tangential cells in the lobula plate then spatially pool signals from four subtypes of T4 and T5 cells, each tuned to one of the four cardinal directions, to extract flow fields associated with particular types of ego motion, generating information that is finally put to use during course control [1].

**Fig 2:**
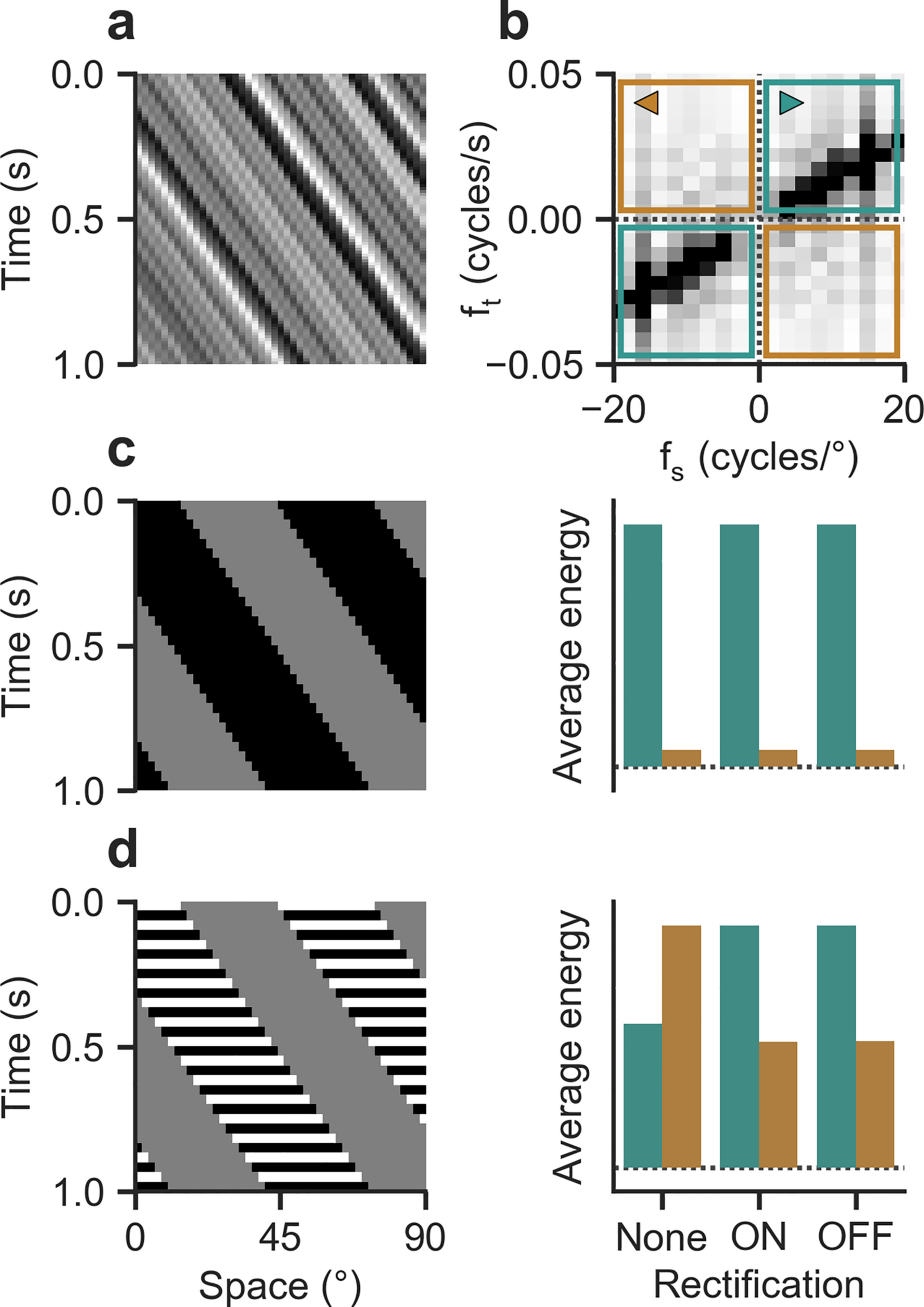
Spectral properties of translating motion stimuli. **a** Space-time plot of a one-dimensional noise pattern moving to the right at constant speed (*v* = 60° s^-1^). **b** Magnitude spectrum of translating noise, with darkness indicating larger frequency contribution. For rigid motion toward the right, components cluster around a line connecting first and third quadrant (in green) via the origin, with slope indicating true velocity. Little magnitude is assigned to frequencies in the quadrants signaling leftward motion (in brown). **c** Left, space-time plot of a discretized square-wave grating (*v* = 60° s^-1^, *λ* = 60°, step width = 2°). Right, quantification of energy indicating right- and leftward motion, respectively. Energy was simply averaged within quadrants color-coded above. Energy distribution is not affected by half-wave rectification (i.e., zeroing either components below or above mean luminance for ON or OFF respectively). **d** Corresponding illustration and quantification for discretized reverse-phi stimulus. Energy distribution reverses for full-wave stimulus but signals veridical direction when rectification is applied (ON or OFF).

This polarized architecture appears particularly interesting given that reverse-phi stimuli seemingly exploit interactions between input of opposite contrast induced by contrast reversals. Such mixed-polarity interactions find a compelling explanation in the Fourier analysis of motion [14,39,40]. Arbitrary patterns rigidly translating along one direction (**Fig 2A**) have a Fourier spectrum whose energy is concentrated along a single line through the origin. This line’s slope and orientation give the true velocity of the stimulus; a model based on Fourier analysis could thus simply average energy in the appropriate quadrants to estimate direction (**Fig 2B**). For a discretized square-wave grating periodically jumping to the right (**Fig 2C**), this energy is confined to the rightward-indicating quadrants. The distribution is not affected by half-wave rectification. If the contrast is reversed with each jump, energy in the opposite direction dominates (**Fig 2D**): the best fitting grating component now travels in an illusory direction, even though veridical motion energy is still present [19]. Critically, for ON- or OFF-rectified reverse-phi stimuli, motion energy remains largely oriented in the true direction of motion. A motion detector in which half-wave rectified ON and OFF signals are processed separately should thus not be susceptible to the illusion.

Previous work has shown that in principle, a detector in which ON and OFF inputs are not directly correlated can explain electrophysiological responses to apparent motion stimuli in which a spatiotemporal mixture of ON and OFF signals elicits reversed responses [4,41]. This presupposes imperfect separation of ON and OFF. Instead of simply half-wave rectifying luminance changes, the input signal retains some sensitivity to absolute luminance in the form of an attenuated DC signal, thus remaining receptive to fluctuations in both directions.

Here, we continued this investigation along two directions. First, we used a periodic reverse-phi stimulus [6,19] to check whether the model would generalize to other forms of the illusion. Of particular interest was the observation that for this class of stimuli, the response reversal only occurred under specific circumstances; namely when velocities were sufficiently low. Psychophysical investigations demonstrate that human perception of reverse-phi strongly depends on factors like eccentricity, spatial proximity, contrast, as well as spatial and temporal frequency [3,10,19,42–49]. This has often found its explanation in a postulated pair of motion detection systems [50], only one of which is susceptible to reverse-phi illusions. The stimulus at hand allowed us to test whether a single model could account for such parameter-driven transitions between illusory and veridical motion direction. Second, we employed neurogenetic manipulation of T4 and T5 units in behaving flies as well as physiology to elucidate the neural circuits underlying these responses.

## Materials and methods

### Fly strains and genetics

For all experiments, we raised *Drosophila melanogaster* on cornmeal-agar medium under standard laboratory conditions (60% humidity, 25°C, 12 h light/12 h dark cycle). Experiments were performed with female flies only. In behavioral assays, we used flies that were between 1 and 3 days old; for electrophysiology, selected flies were 4–16 h post-eclosion. Lobula plate tangential cell recordings and certain behavioral experiments were performed on Canton S wild-type flies. For behavioral experiments on T4/T5 block flies, we pursued a standard genetic control strategy: T4 and T5 cells were simultaneously targeted with a highly specific splitGal4 line (R59E08-AD; R42F06-DBD) [26,33] that we crossed to either Canton S flies (yielding the T4/T5 control genotype) or pJFRC100-20XUAS-TTS-Shibire-ts1 (*shibire*^*ts*^) flies [51,52] (yielding the T4/T5 block genotype). The *shibire*^*ts*^ (“shi”) control genotype was generated by crossing the *shibire*^*ts*^ line to Canton S flies. When silencing T4 or T5 in isolation, we made use of the light chain of tetanus toxin (TNT) [53] to remain consistent with earlier work [27,33]. Block and control genotypes were generated from T4-Gal4 (VT37588), T5-Gal4 (R42H07), and UAS-TNT-E according to the scheme described above. For imaging experiments, we used lexAop-GCaMP6m flies [54] recombined with the lexA-VT50384 line expressing predominantly in T4/T5 axon terminals of layer 3 in the lobula plate as previously described [55].

### Behavioral experiments

We performed treadmill experiments as described previously [33]. Briefly, flies were tethered under cold anesthesia and placed on polyurethane balls suspended in a constant air stream from below. Flies were surrounded by three LCD screens that covered a substantial portion of the animals’ visual field (approximately 270° in azimuth and 120° in elevation). Screens refreshed at either 120 or 144 Hz, depending on the specific set-up; at no point could we observe any behavioral difference between the two update rates. As a result of utilizing high-definition displays, resulting retinal pixel size was far below the resolution limit of the fruit fly. Using the game engine Panda3D, we generated and projected visual patterns online. All stimuli were perspective-corrected to simulate a surround that would appear cylindrical from the viewpoint of the fly. Two high-speed cameras tracked ball movements in real time and at a resolution of 4 kHz that was down-sampled to 20 Hz for analysis. We controlled air temperature in the vicinity of flies by means of a closed loop thermal regulation system. For all experiments, including genetic controls, initial temperature was set to 25°C and then raised to 34°C within 10 min in order to trigger the thermogenetic effector *shibire*^*ts*^ as well as to ensure constant forward movement of the fly.

We selected flies post-hoc based on both their mean walking speed, which we required to be above 4 mm s^-1^, and their general turning tendency, which we required to be stable and close to zero in the temporal average. For each fly, we selected a continuous range of 40 out of 70 trials (corresponding to 47 out of 82 min of experiment) for which these conditions held. Around 10% of all animals were discarded based on our criteria.

Our visual stimuli were modeled after previous work [6] (see illustrations in **Fig 2**). Briefly, we presented whole-field square-wave gratings with spatial wavelengths of *λ* = 30° or 90°. Alternating stripes were set to an intermediate luminance value. Other stripes were either as dark or as bright as possible, depending on current contrast state, yielding an instantaneous Michelson contrast of 50%. We fixed the step distance at 4° which gave us the discrete movement typical for phi-type motion stimuli. Stimulus velocity then determined at what frequency the grating would update (velocity *v* over step width). Each trial lasted 3.5 s and the grating moved between 1.0 and 2.0 s. For what we call the phi condition, we kept the brightness of non-gray bars constant and randomly set them to dark or bright on each trial. For the reverse-phi condition, non-gray luminance flipped between dark and bright on each step. We showed mirrored versions of each stimulus, with the pattern moving toward either the left or the right, and subtracted responses during analysis to eliminate possible turning bias of the fly. Stimuli were presented at a total of 5 velocity conditions (*v* = 16, 32, 64, 128, or 256° s^-1^) and in randomized order. When motion and flicker components were varied independently, we tested all 25 combinations of 5 update frequencies (*f* = 0, 8, 16, 32, 64 Hz); the diagonal of this matrix corresponds exactly to the previously used pattern velocities. All other parameters were as before. We additionally implemented a velocity condition in which the stimulus was exactly synchronized with the refresh rate of our 144 Hz screens, resulting in the maximum attainable velocity of 576° s^-1^. Further analysis was based on trial-averaged traces. To generate summarized responses, we took the mean of turning velocity between 1.5 and 3.0 s after trial onset. All behavioral evaluations were written in Python using the NumPy, SciPy, pandas, and Numba libraries.

### Electrophysiology

Our *in-vivo* patch-clamp recordings from lobula plate tangential cells of the horizontal and vertical systems were performed as described previously [33]. Cell identity was determined by presenting vertical and horizontal moving gratings. Signals were captured at 2 kHz. During physiological experiments, we stimulated flies using a custom-designed cylindrical LED arena that covered 180° in azimuth and 90° in elevation. This device offered a spatial resolution of approximately 1.5° per image pixel. LEDs flickered at more than 1 kHz and had a maximum luminance of 80 cd m^-2^.

We evaluated data offline by averaging across 2–5 stimulus repetitions. Stimulus presentation was randomized. Phi and reverse-phi stimuli had the same structure as the behavioral ones, with parameters fixed at *λ* = 45°, *v* = 128° s^-1^, and step width = 6°. Depending on cell type, we set the grating orientation to either horizontal or vertical. Preferred direction was defined as the direction that depolarized the unit under phi conditions. We generated summary statistics by averaging across the first two seconds of stimulus presentation. Cells that showed an average depolarization of below 6 mV for the preferred direction phi condition were discarded.

### Calcium imaging

Calcium imaging in T4 and T5 dendrites was performed as described previously [38,55]. Briefly, we acquired images at an isotropic resolution of 64 pixels and 7.51 Hz. Responsiveness was assessed by sweeping gratings before the experimental protocol started. Stimuli were projected at 180 Hz and significantly above the fly’s spatial resolution limit on a custom-designed DLP projector-based arena which covered approximately 180° in azimuth and 105° in elevation of the animal’s visual field. Stimuli had identical properties as the ones used throughout our behavioral experiments and were rendered using custom software written in Python and Panda3D. Mean luminance for the reverse-phi stimuli was 55 cd m^-2^.

During data analysis, we automatically registered images based on linearly smoothed cross-correlation. Regions of interest were selected by hand and through visual inspection of calcium activity. We did not attempt to identify individual terminals; instead, we averaged activity across full neuropils (medulla for T4 and lobula for T5). To estimate ΔF/F reliably, we determined the minima of our calcium signals within a 15 s sliding window. Finally, we subtracted and divided the signals by the resulting trace. We wrote all physiological evaluations in Python using the NumPy, SciPy, pandas, and Numba libraries.

### Modeling

We modeled elementary motion detectors in accordance with previous work [4,33]. Specifically, we simulated a one-dimensional array of 60 units, each receiving two inputs separated by 4° in visual space. For non-rectified detectors, input signals were high-pass filtered with τ_HP_ = 250 ms, asymmetrically low-pass filtered with τ_LP_ = 50 ms, and multiplied. This was done twice in a mirror-symmetrical fashion. Resulting output was subtracted and summed across detectors. For the rectified unit, a high-pass filtered signal and 10% of unfiltered input (that is, a DC component) were summed, followed by half-wave rectification at zero which yielded either a positively valued ON or a positively valued OFF signal. Downstream processing was equivalent to the non-rectified unit, with ON and OFF detectors being summed post-subtraction. Where specified, we modulated filter time constants; otherwise, both detector models shared temporal parameters. When analyzing responses of subunits, we set the weight of the other pathway to zero. All filters were RC filters of first order and integrated using the Euler Forward method. We carried out simulations at a time step of 1 ms, which was small compared to all time constants and velocities involved, and at a spatial resolution equivalent to one receptor distance. No spatial blurring was applied before feeding signals into the detectors.

Note that we did not refit parameters for filters and DC level. All settings are equal to those in previous work [4] with the exception of rectification offset and preferred-null imbalance which we chose to remove for reasons of parsimony. Stimuli were constructed analogously to the behavioral experiments but restricted to one spatial dimension. Each presentation ran for 10 s (with the stimulus moving between 0.5 and 9.5 s). The step width was set to one receptor distance; spatial wavelength varied and is given in the corresponding figure legend. We simulated 20 velocities on a logarithmic scale from 1 to 1000° s^-1^ except for experiments where we varied flicker and motion frequencies independently. For the out-of-phase control stimulus, phases of the two update cycles were shifted by half of a period to temporally decouple flicker and motion. Luminance values we set to 1.05/1.3/1.55 (dark/intermediate/bright). Finally, we generated response estimates by averaging output across time and space. All simulations were written in Python using the NumPy, SciPy, and Numba libraries.

### Statistics

Unless stated otherwise, error bars and shadings indicate 95% confidence intervals around the mean, computed through an iterative bootstrapping procedure based on 1,000 re-samplings. These intervals allow hypothesis testing at an alpha level of 0.05. Where appropriate, we additionally established significance at equal alpha level by applying a Bonferroni-corrected two-sided Student’s t-test with variances assumed to be unequal. For blocking experiments, results were deemed significant overall if and only if block flies significantly differed from both controls. Sample sizes were predetermined without power calculations and are in line with established research [5,33,56–58]. We did not blind experimenters to genotype conditions.

### Code availability

Data and code to generate all figures may be found online (https://github.com/borstlab/reversephi_paper).

## Results

### T4 and T5 are necessary for all aspects of reverse-phi behavior

Our initial objective was to investigate whether a combination of motion detectors sensitive to either positive (ON) or negative (OFF) brightness changes is sufficient to explain behavior observed for an ON-OFF interaction stimulus like reverse-phi. To this end, we conditionally silenced the synaptic activity of local ON and OFF motion detector cells T4 and T5 using Gal4-targeted expression of the temperature-sensitive dynamin allele *shibire*^*ts*^ [26,51] and tested if turning responses for phi or reverse-phi patterns were affected at restrictive temperatures.

**Fig 3:**
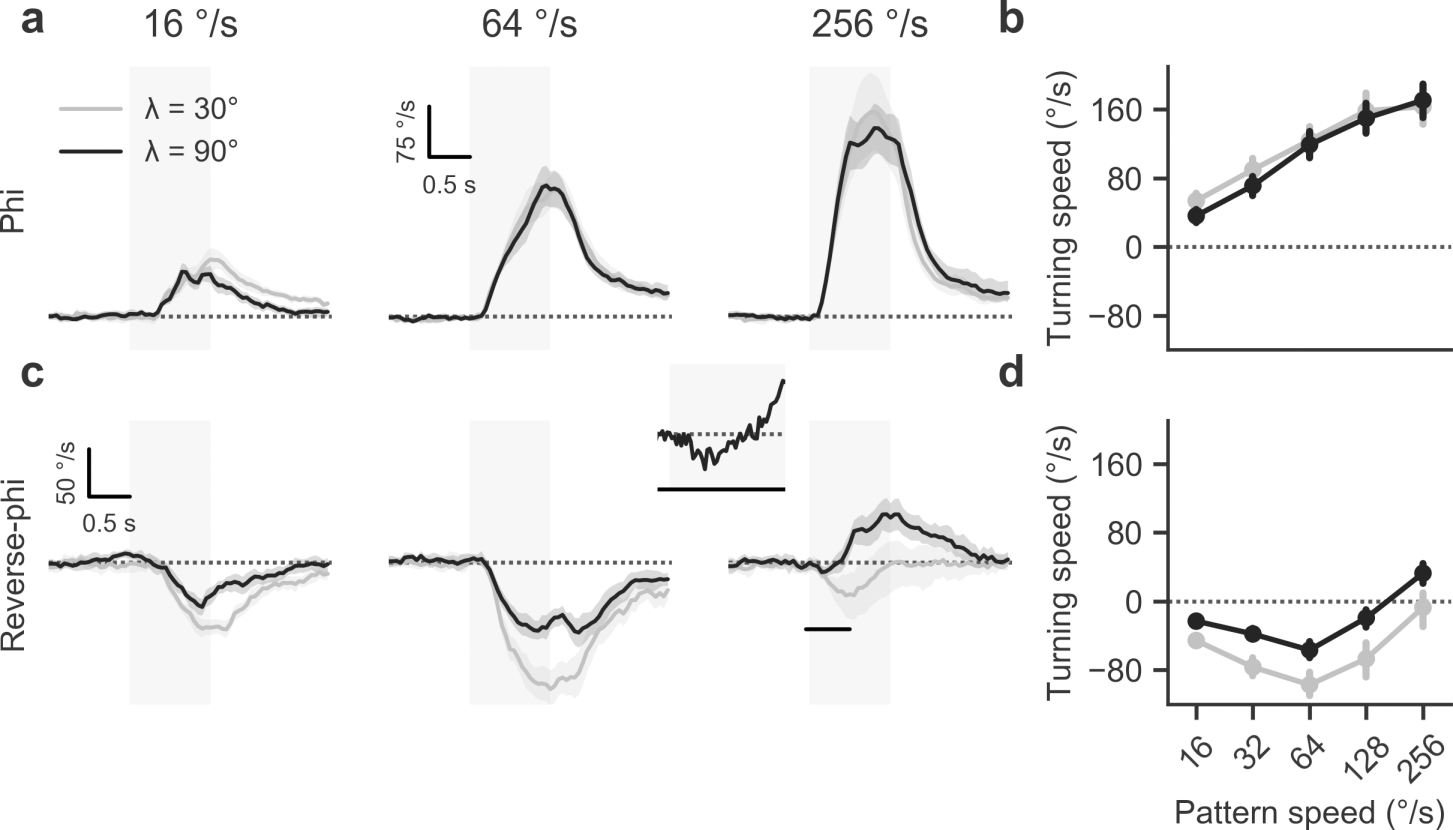
Reverse-phi responses in tethered walking flies. **a** Average turning response for phi condition at three different stimulus velocities and two spatial wavelengths, indicated by panel title and line color. Data are from T4/T5 control flies. Control flies (*N* = 12) rotate syndirectionally with stimulus. Gray shaded area indicates stimulation period. **b** Summary statistics for phi condition (average from 1.5 to 3.0 s after trial onset). **c** Average turning response for reverse-phi condition. Control responses (*N* = 10) are inverted at low velocities. At high velocities and depending on spatial wavelength, the optomotor response reverses again. The inset depicts the first 500 ms (see black line in main panel) of the response to stimulation with a pattern velocity of 256° s^-1^ (*λ* = 90°), highlighting temporally biphasic dynamics. **d** Summary statistics for reverse-phi condition. Shaded areas around curves and bars around points indicate 95% confidence intervals. See Materials and Methods for details on behavioral experiments.

Previous work on *Drosophila* [6] had shown that in flight, flies respond vigorously to both types of motion stimuli by steering along with or opposed to pattern direction, respectively. We first aimed to replicate the basic phenomenology and to extend the findings to walking *Drosophila*. When we presented control flies with a discretized phi motion stimulus (**Fig 2C**), comparable to ordinary grating motion, we observed strong turning responses whose direction matched that of pattern motion (**Fig 3A**). As expected for such optomotor responses in the fly, the amplitude of turning was strongly modulated by pattern velocity (**Fig 3B**). In the range tested, responses rose steadily and approximately linearly. Additionally, we tested the effect of modulating the spatial wavelength of the pattern. For phi motion, differences were marginal. Given that insect motion detectors are generally tuned to temporal frequency as opposed to velocity [1], we expected turning to decline at high velocities, with the position of the peak depending on pattern wavelength. This was not borne out by data, presumably because this would require velocities not achievable on our stimulation device [6].

In a subsequent series of experiments, we added contrast reversals to the phi motion stimulus (**Fig 2D**) which gave reverse-phi motion. Wild-type behavior from previous flight experiments was largely recapitulated: flies steered in the opposite direction of pattern motion for a range of low to medium velocities (**Fig 3C and 3D**). As observed before, the overall magnitude of turning responses was reduced compared to the standard phi condition. Tuning curves were shifted toward lower pattern velocities. Critically, for large wavelengths, reverse-phi turning peaked at around 64° s^-1^, decreased in amplitude, and finally became weakly but robustly syndirectional with stimulus motion at high velocities (**Fig 3D**). For the lower wavelength we tested, response magnitude was generally larger and did not re-invert at high velocities. Re-inverted turning tendency exhibited biphasic dynamics where flies would first turn against pattern motion and then reverse within hundreds of milliseconds, now rotating syndirectionally with the stimulus (inset in **Fig 3C**). This particular behavior matched flight responses [6] and rendered us confident in sensory mechanisms upstream of motor command generation being responsible for the observed re-inversion.

**Fig 4:**
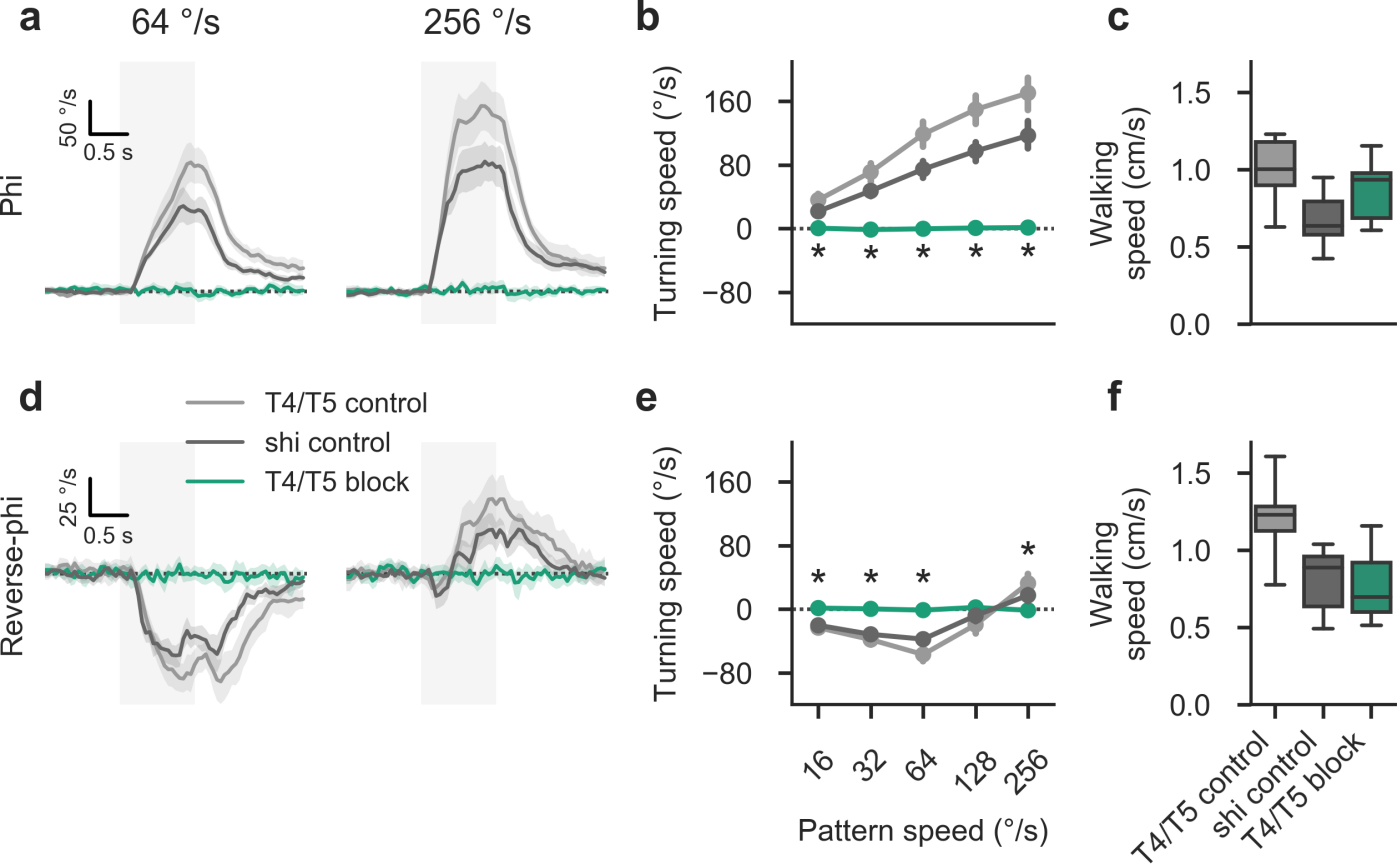
Local motion-sensitive cells T4 and T5 are required for reverse-phi responses in walking flies. **a** Average turning response for phi condition at two different stimulus velocities, indicated by panel title (*λ* = 90° and step width = 4° throughout figure). Here, control flies (*N* = 12/12 for *shibire*^*ts*^ and T4/T5 controls) turn with the direction of the stimulus. This behavior is abolished in T4/T5 block flies (*N* = 11). Gray shaded area indicates stimulation period. **b** Summary statistics for phi condition (average from 1.5 to 3.0 s after trial onset). **c** Walking speed statistics (averaged as before) for phi condition. No genotype exhibits impaired locomotion. **d** Average turning response for reverse-phi condition. Control responses (*N* = 11/10 for *shibire*^*ts*^ and T4/T5 controls) are inverted at low velocities. At high velocities, re-inversion occurs. Turning is fully abolished in T4/T5 block flies (*N* = 10). **e** Summary statistics for reverse-phi condition. **f** Walking speed statistics for reverse-phi condition. Shaded areas around curves and bars around points indicate 95% confidence intervals. Asterisks mark significant differences between T4/T5 block flies and both controls (Student’s t-test, *P* < 0.001). Note that whiskers show the full sample range. See Materials and Methods for details on behavioral experiments and statistics.

Turning responses for phi motion were fully abolished when we examined flies in which we had selectively silenced T4 and T5 (**Fig 4A and 4B**). This was in line with expectations and previous results [20,33,56]. We could rule out motor dysfunction as an explanation for the substantial effects given that the forward walking speed of T4/T5 block flies remained at control level (**Fig 4C**). Finally, when we silenced T4 and T5 activity, all reverse-phi turning responses, including high velocity re-inversion, were eliminated (**Fig 4D and 4E**). This was again not accounted for by motor deficits (**Fig 4F**). In summary, we found ON- and OFF-specific local motion detectors T4 and T5 to be necessary for all aspects of contrast-induced motion reversals, strongly suggesting that no secondary pathways play a role in generating these responses.

### Tangential cells exhibit partial reverse-phi sensitivity

We next explored whether large-field motion-sensitive tangential cells in the lobula plate show reverse-phi responses that correspond to observed behavior. Receptive fields of tangential cells match various types of optic flow and are thought to directly control compensatory steering behavior such as the optomotor reflex [1,59]. Here, we sampled from cells of both the horizontally and vertically sensitive systems [60,61], which we pooled given that no functional differences were observed for this assay. Critically, T4 and T5 provide the main excitatory and, via LPi interneurons, inhibitory input to these units [62,63]. If these input elements are inactivated, tangential cells become fully unresponsive to motion [64]. A previous study had used dendritic calcium imaging to examine their response properties for reverse-phi stimuli. This did not permit disambiguation of zero and hyperpolarizing responses and was limited in temporal resolution [6]. As an extension, we therefore performed *in vivo* patch-clamp recordings from tangential cells in immobilized flies to further characterize excitatory and inhibitory inputs originating from T4 and T5.

**Fig 5:**
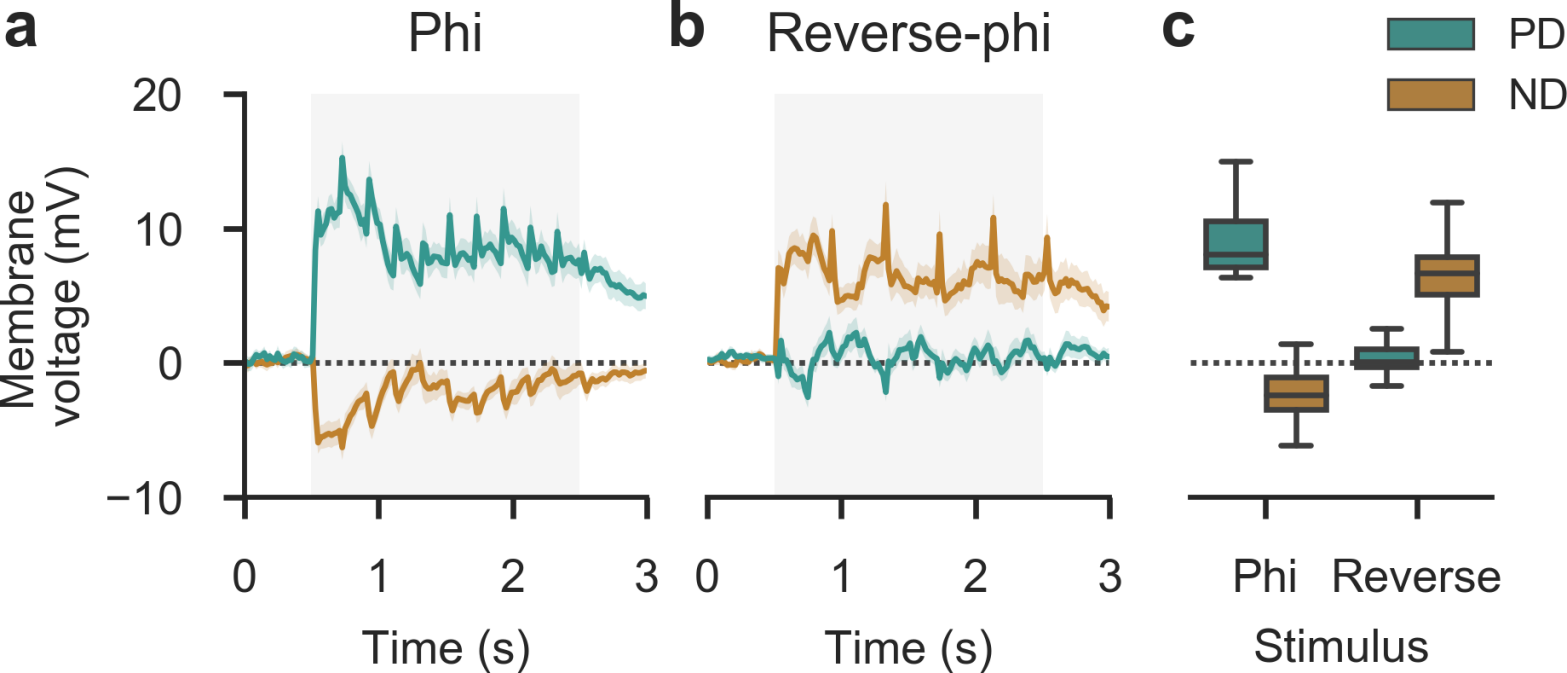
Motion-sensitive tangential cells show selectively inverted responses upon reverse-phi stimulation. **a-b** Average membrane potential of lobula plate tangential cells (*N* = 23, pooled across 8 horizontal and 15 vertical system cells, with accordingly oriented direction of movement) in wild-type flies. Responses were recorded at 2 kHz and down-sampled to 50 Hz for presentation. Gray shaded area indicates stimulation period (*λ* = 45°, *v* = 128° s^-1^, step width = 6°). Shading around traces shows bootstrapped 95% confidence intervals. **a** Phi stimulation. Cells depolarize for motion in preferred direction (PD) and hyperpolarize weakly for motion in the opposite null direction (ND). **b** Reverse-phi stimulation. The PD response is abolished, whereas the ND response inverts. **c** Summary statistics (averaged across initial 2 s post-onset). Note that whiskers show the full sample range. See Materials and Methods for details on electrophysiology experiments.

Tangential cells showed strong sustained depolarization when flies were stimulated with phi motion along the preferred direction of the cell at intermediate velocity (*λ* = 45°, *v* = 128° s^-1^, step width = 6°). In turn, when movement in the null direction was displayed, we observed weaker but substantial hyperpolarization (**Fig 5A and 5C**). This was in agreement with established motion-sensitive properties of these elements. As predicted, under reverse-phi stimulation in null direction tangential cells now depolarized, albeit with decreased amplitude (**Fig 5B and 5C**). This matched reduced behavioral responses for reverse-phi stimuli, lending further credence to the hypothesis that these cells control steering. Interestingly, preferred direction stimuli did not elicit detectable voltage deflections. This may be due to synaptic input being insufficient to drive the inhibitory interneurons that mediate hyperpolarization [63]. In summary, we found that tangential cells display at least partial voltage response inversion under reverse-phi stimulation. Given their input structure and taken together with our behavioral findings, this implies T4 and T5 are jointly necessary and sufficient to generate these responses.

### A simple detector model predicts reverse-phi responses in detail

Next, we set out to determine whether a biologically plausible detector model can account for the responses evoked by this particular form of movement illusion. A simple algorithmic model, the Hassenstein-Reichardt detector [9], has proven particularly powerful at explaining motion detection-related phenomena in insects. It consists of two visual inputs that are asymmetrically filtered in time, non-linearly combined through, for instance, multiplication, and finally subtracted from the output of a spatially mirrored operation to generate bidirectional motion sensitivity [1,9,65].

**Fig 6:**
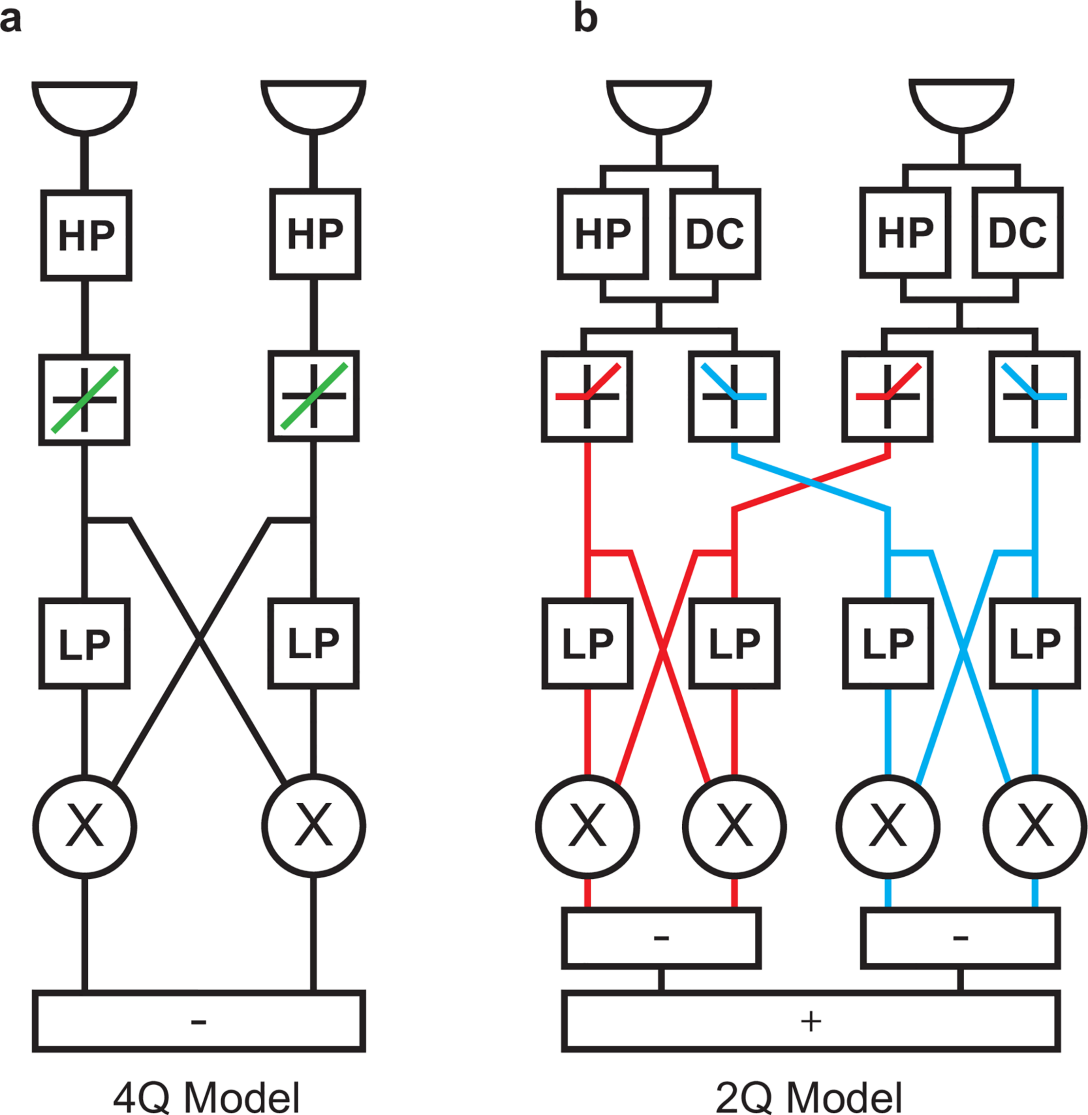
Architecture of four- and two-quadrant motion detection models. **a** Signal flow for a single four-quadrant, non-rectifying elementary motion detector (HP: first-order high-pass filter, LP: first-order low-pass filter, X: multiplication). Quadrants are possible combinations of positive (ON) and negative (OFF) signal contrast. Properties of rectification are displayed as schematic transfer functions (non-rectified in green, ON in red, OFF in blue). If no rectification between ON and OFF occurs, all four possible correlations (ON-ON, ON-OFF, OFF-ON, OFF-OFF) are directly computed. **b** Signal flow for two-quadrant, rectified elementary motion detector (DC: direct unfiltered input component). Here, signals of equal sign (quadrants ON-ON and OFF-OFF) are correlated.

In such elementary motion detectors (**Fig 6A**), reverse-phi responses find a simple explanation in sign-correct multiplication. Two positive or two negative signals, corresponding to pure ON or OFF signals, produce positive output. If inputs are mixed, like for the ON-OFF interactions of our reverse-phi stimulus, rules of multiplication lead to negative output. Indeed, an early observation of the reverse-phi phenomenon in insects was instrumental in deriving this motion detection scheme [9]. Sign-correct multiplication may be realized on a neuronal level by splitting the signal into ON and OFF components, then computing the four possible quadrants (ON-ON, ON-OFF, OFF-ON, and OFF-OFF) separately, and finally summing them with appropriate signs.

Given overwhelming experimental evidence that motion detection in the fly visual system is based on a combination of polarity-specific ON- and OFF-sensitive units [2,20,27,30,33,56,64], an elaborated motion detection scheme has gained in importance: the two-quadrant (2Q) motion detector [4] depicted in **Fig 6B**. Here, multiplication is preceded by differentiation, as approximated by a high-pass filter, and subsequent half-wave rectification. Only two possible combinations of inputs are then correlated, corresponding to the ON-ON and OFF-OFF quadrants mentioned above. This stands in contradistinction to a four-quadrant (4Q) scheme which is mathematically equivalent to the simple, non-rectified Hassenstein-Reichardt detector (**Fig 6A**). The 2Q detector has the advantage of being mappable onto neural architecture: the ON-ON subunit models the T4 pathway, the OFF-OFF subunit the T5 pathway. For apparent motion stimuli, it was possible to explain reversed responses to interactions between ON and OFF by introducing a tonic luminance component (DC) added to high-pass filtered input before the rectification stage. In effect, this renders the half-wave rectification of inputs imperfect; some residual sensitivity to both contrast polarities is preserved even after pathway splitting. It was not clear, however, whether this approach would generalize to other stimuli.

**Fig 7:**
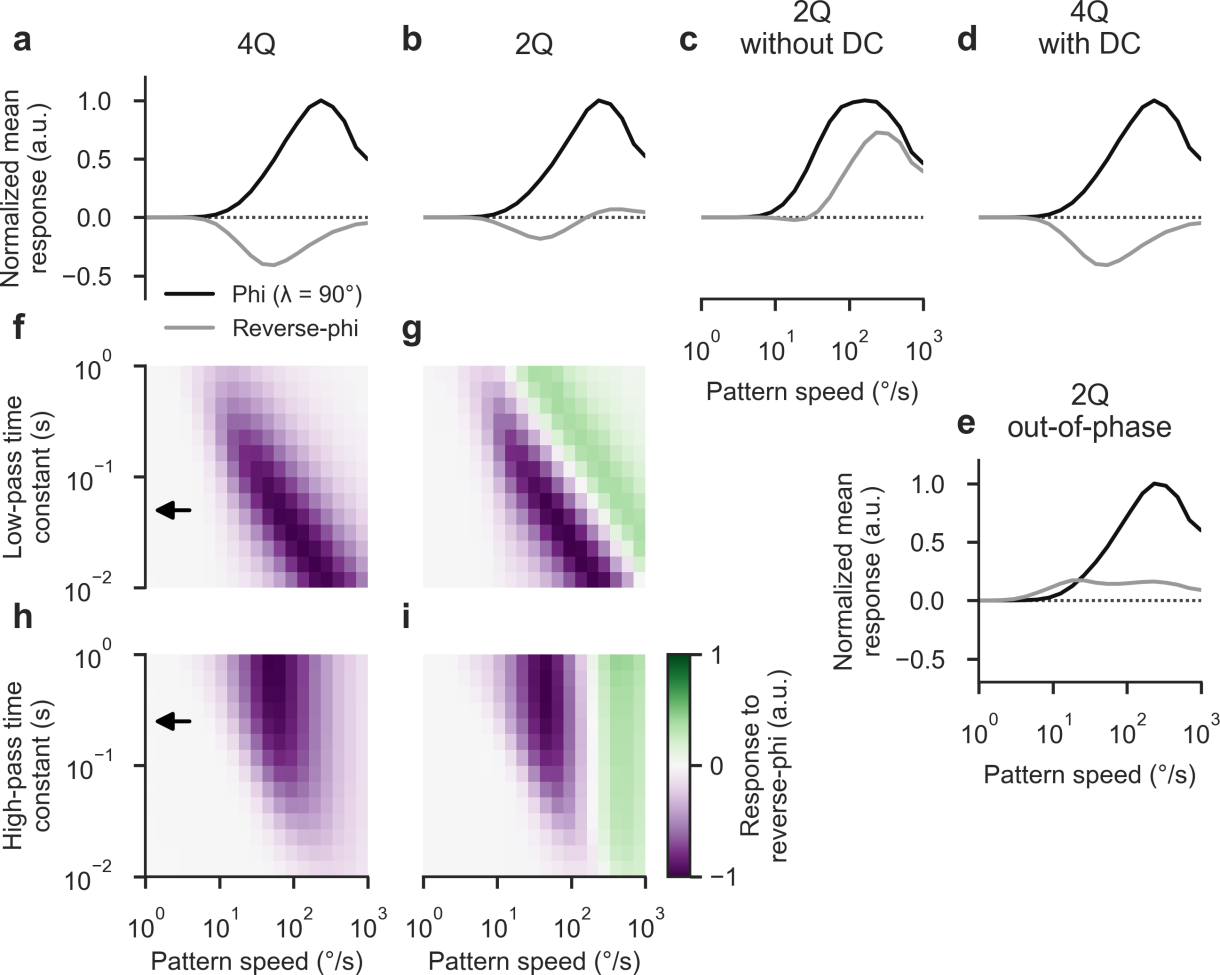
A two-quadrant model admitting constant luminance accounts for behavioral and neural responses. **a-b** Velocity tuning of two model architectures (4Q: non-rectified four-quadrant detector; 2Q: rectified two-quadrant detector with constant luminance contribution, DC). All detectors had identical base parameters (τ_HP_ = 250 ms; τ_LP_ = 50 ms; DC = 0 or 10% for 4Q or 2Q, respectively; receptor distance = 4°). Simulations were carried out at time steps of 1.0 ms over 10 s of stimulation per velocity. Lines represent time- and space-averaged responses of 60 detectors covering 240° of visual space, normalized to +/-1 maximum/minimum per panel. Black lines: Responses to phi stimulation across 20 velocities on logarithmic scale (*λ* = 90°, step width = 4°). Gray lines: Responses to corresponding reverse-phi stimulation. Only the rectified two-quadrant model replicates both response reversal and re-inversion at high velocities, as indicated by experimental results. **c** Velocity tuning for rectified 2Q detector with DC set to zero. Reverse-phi response now indicates veridical direction across full range. **d** Velocity tuning for 4Q detector with DC level set to 10%. **e** Velocity tuning for 2Q detector when flicker and motion updates are out of phase. **f-i** Parameter scans for low- and high-pass time constants of 4Q and 2Q detectors. Arrows indicate default setting. The respective other time constant was kept at its default value. Qualitative tuning features appear to not depend on choice of parameters. See Materials and Methods for further details on simulations.

We tested both detector types to assess whether they would capture the particular features of our behavioral data set, using identical stimuli as in our experiments. First, the 4Q detector qualitatively reproduced velocity tuning for phi stimuli, complete response inversion for reverse-phi stimuli, and a response peak difference between the two conditions (**Fig 7A**). As in our behavioral data, the tuning curve was shifted toward lower velocities for reverse-phi stimulation and had consistently lower response amplitudes. Critically, though, and as noted before [6], it failed to show the typical re-inversion for high pattern velocities. Instead, responses smoothly approximated the zero line from below.

Second, the 2Q detector with DC could successfully account for all major qualitative features of reverse-phi responses in walking or flying *Drosophila* (**Fig 7B**). Output for reverse-phi was inverted, with the peak shifted toward lower velocities when compared to phi output. Most importantly, at the high end of the velocity spectrum responses did re-invert and then approached zero from above. This was in agreement with our data and previous investigations in flight [6], even if the velocity optima did not quite match our findings (**Fig 3**). Response strength was substantially reduced for reverse-phi stimulation, at a factor exceeding expectations from behavioral findings. However, the mapping from T4/T5 output to behavioral response is likely to be compressive which could account for the discrepancy [28,66]. Third, we performed multiple controls to assess the specificity of the observed inversion. To assess whether tonic luminance sensitivity was the critical determinant for reverse-phi responses, we disabled the DC contribution. While, as expected, phi responses were still predicted correctly, reverse-phi output did now not flip sign (**Fig 7C**). We propose that for a strictly ON-OFF separated system such as the DC-less 2Q detector, the reverse-phi stimulus simply appears as an interrupted grating with greater step width, which explains the direction selectivity of responses as well as the shifted velocity optimum. When we added a DC term to the standard 4Q detector, no qualitative change in tuning properties occurred which suggests that the conjunction of DC and rectification plays a critical role in shaping reverse-phi output (**Fig 7D**). Moreover, an out-of-phase stimulus in which flicker and motion updates were not synchronized elicited reduced but consistently positive responses (**Fig 7E**). This matched the uniformly syndirectional turning reported previously for this variant of the pattern [6] and excluded unspecific interference between flicker and motion as an explanation for inverted 2Q responses. Interestingly, behavioral data from flying flies exhibited a modest reduction in wing-beat amplitude for intermediate velocities. The 2Q model predicts this particular tuning characteristic.

Finally, we evaluated the relationship between filter settings and reverse-phi responses. For all previous model experiments, time constants were set to values proposed previously [4]. To gauge whether other parameter combinations would elicit response re-inversion for the 4Q architecture, we systematically varied either low- or high-pass time constant while keeping the respective other at its default value. As expected, modulating the low-pass filter had the effect of shifting the velocity optimum from low to high for large to small time constants in both the 4Q (**Fig 7F**) and the 2Q detector (**Fig 7G**). For no time constant did the 4Q response re-invert. For the 2Q detector, the peak of re-inversion shifted along with the velocity peak of initial response reversal. At the extreme end of the time constant spectrum, responses were globally attenuated as expected from a low-pass filter. Trivially, the opposite was found when modifying the high-pass time constant: for small time constants, responses vanished in both 4Q (**Fig 7H**) and 2Q (**Fig 7I**) models. Peak positions were not affected by these manipulations. Critically, 4Q responses remained fully inverted across the whole range while the 2Q detector consistently showed bilobed velocity tuning.

**Fig 8:**
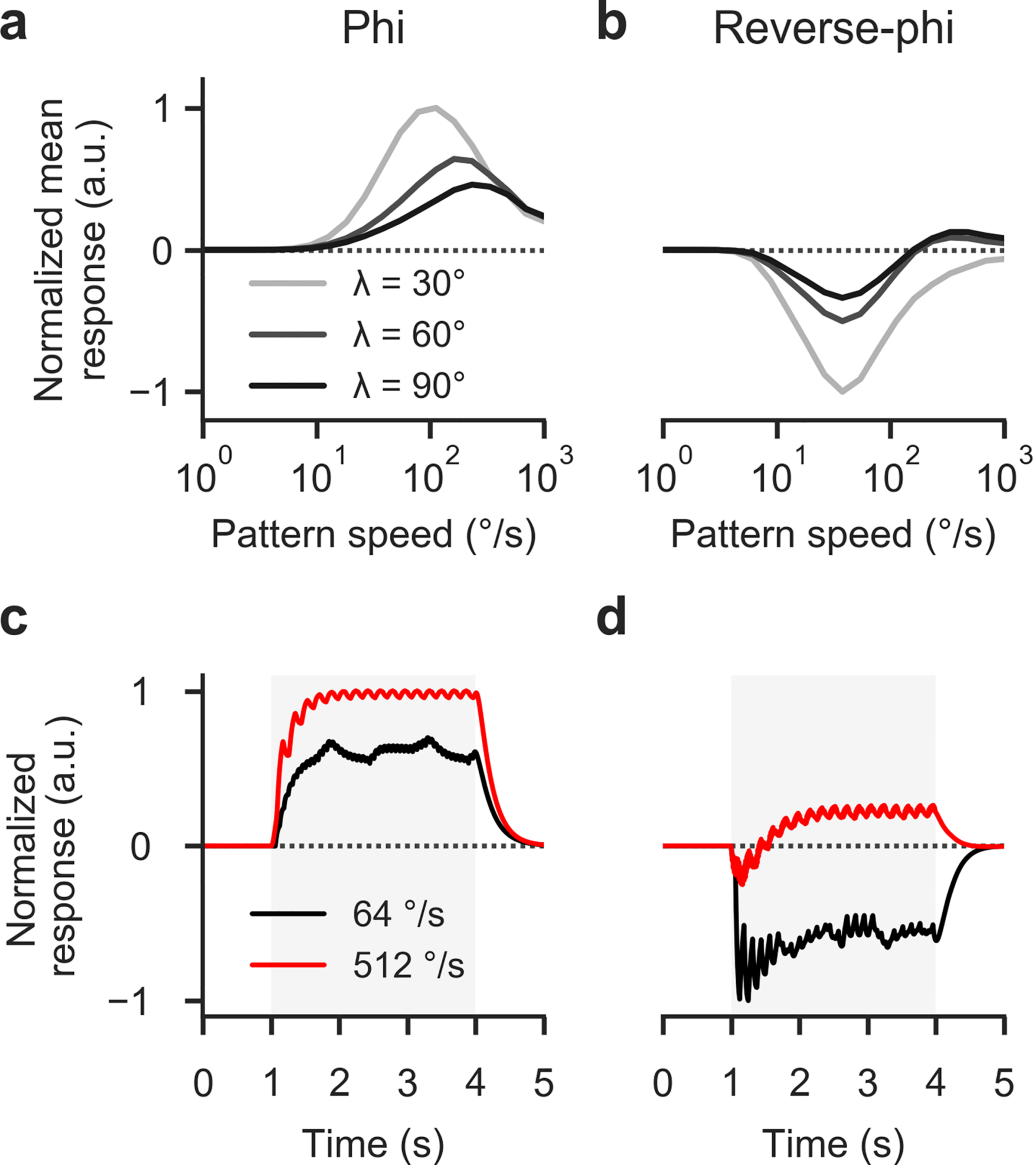
Detailed spatiotemporal tuning of 2Q detector. **a** Phi velocity tuning curves of 2Q detector for different spatial wavelengths. Response optima shift toward higher velocities with increased *λ* as predicted for Reichardt-type motion detectors. **b** Velocity tuning for reverse-phi condition. Here, response peaks remain fixed. Spatial wavelength affects magnitude of optimal response and modulates inversion at high pattern speeds, in line with behavioral findings. **c** Temporally resolved response of a summed array of 2Q detectors in response to phi motion (*λ* = 90°). Traces were lightly low-pass filtered (τ = 200 ms) to approximate temporal integration during visuomotor transformation. Regardless of velocity, the steady-state response indicates veridical motion. **d** Resolved 2Q response to reverse-phi stimulation. At low velocities the response is consistently inverted; for fast velocities, a brief inversion is rapidly followed by positive steady-state output (compare behavioral data in **Fig 3C**). All parameters are set to their default values. Note that we normalize response peaks per panel. See Materials and Methods for further details on simulations.

Behavioral findings had shown spatial wavelength to have a strong effect on reverse-phi responses [6] (**Fig 3**). We therefore tested whether the 2Q detector would be able to explain the specific phenomenology. When stimulated with reverse-phi patterns of differing wavelength, phi responses showed the temporal frequency tuning typical for Hassenstein-Reichardt correlators: increases in wavelength proportionally shifted the position of the peak to larger velocities such that the ratio between velocity and wavelength remained constant (**Fig 8A**). For reverse-phi stimulation, we discovered a different pattern (**Fig 8B**). Peak position was fixed, indicating tuning to velocity or, here equivalently, update frequency. Moreover, with increasing wavelength, reversed responses diminished and re-inversion appeared. This mirrored behavioral phenomenology (**Fig 3**).

Remarkably, the 2Q model faithfully predicted specific dynamics for re-inverted responses. In the case of phi motion, the time-resolved output of the summed detector array quickly approached a steady-state that critically depended on pattern speed (**Fig 8C**). For slow reverse-phi velocities, instantaneous detector output was negative and remained so across the full stimulation period (**Fig 8D**). However, at fast velocities the response became biphasic. Over the course of hundreds of milliseconds the response went through a negative transient and finally reached a positive steady-state. This closely matched behavioral phenomenology in flying and walking flies (**Fig 3C**; [6]). The effect was previously suggested to rely on rapid neural adaptation, but a simple 2Q detector appears capable of reproducing these complex dynamics even in the absence of any such mechanism.

**Fig 9:**
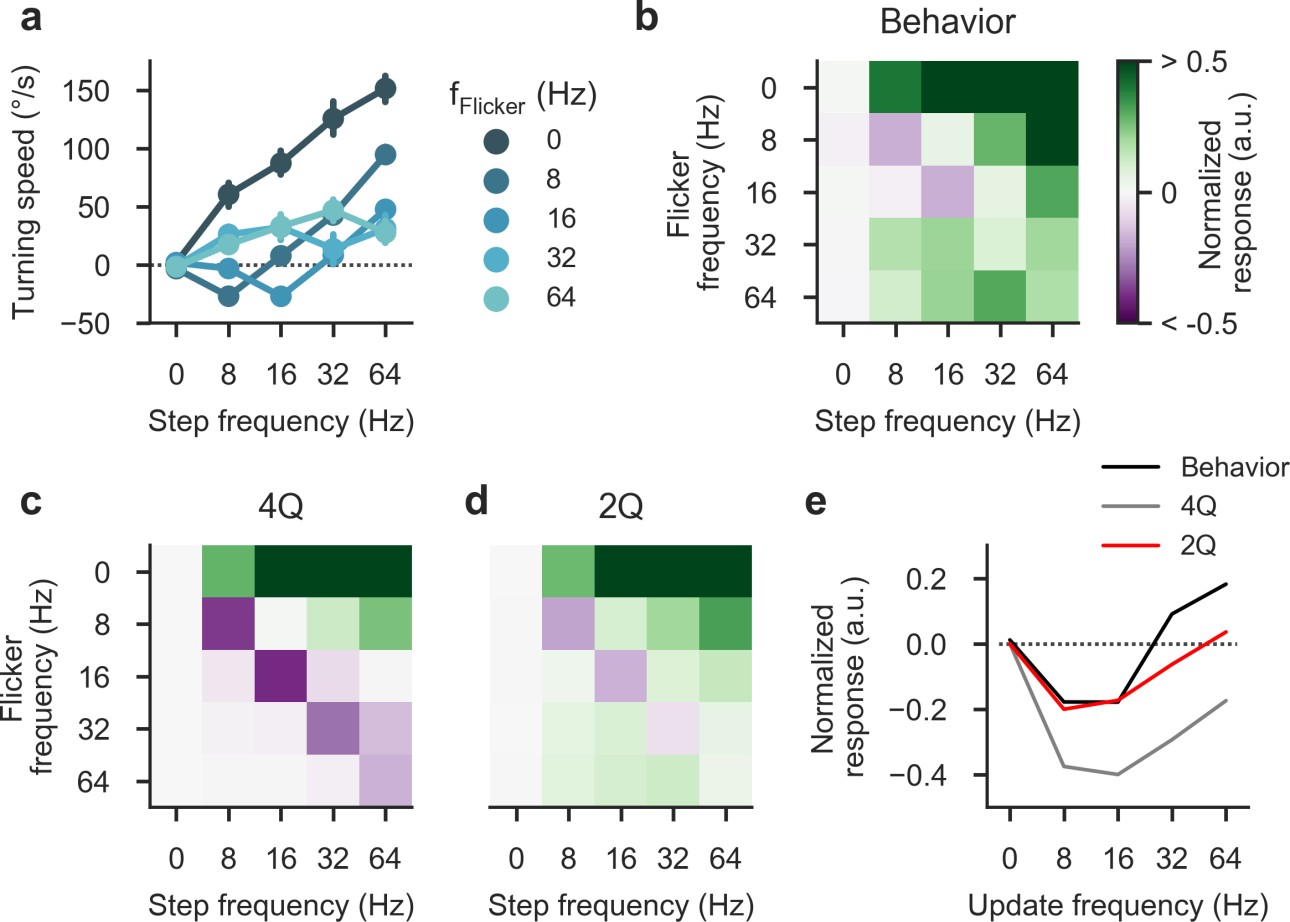
Decoupling flicker and motion components. **a** Turning responses of wild-type flies (*N* = 11) for phi-type stimuli in which flicker and motion frequencies were varied independently (equivalent stimulus velocities are obtained through multiplication by the spatial step width of 4°; *λ* = 90°). Response sign and magnitude are a complex function of both parameters. **b** Response matrix for normalized data from **a** (with maximum set to 1.0). The first row and the diagonal correspond exactly to previously used phi and reverse-phi stimuli, respectively. Note that values are clipped at 0.5. **c** Response matrix for a four-quadrant detector using the same stimulus parameters as in the behavioral experiment. Critically, this model does not predict positive responses below the diagonal. **d** Response matrix for a two-quadrant detector. Crucial features like positive below-diagonal responses are captured faithfully. **e** Direct comparison of diagonal values equivalent to the standard reverse-phi stimulus. Bars around points indicate 95% confidence intervals. See Materials and Methods for details on behavioral experiments and statistics.

We next explored a set of reverse-phi stimuli in which update frequencies for motion and flicker were decoupled. Standard reverse-phi patterns synchronize the two components such that contrast reversals exactly align with discretized motion. A previous study had shown that in flying behavior, varying the rates independently results in a complex response pattern that is not well predicted by standard 4Q motion detection schemes [6]. We repeated these experiments with walking flies and found comparable turning tendencies that were jointly determined by flicker and motion frequency (**Fig 9A**). When evaluating mean responses in matrix form, it becomes apparent that sign and magnitude depend on both parameters; in particular, we observed strong syndirectional rotation for all off-diagonal stimuli in which the two frequencies diverged (**Fig 9B**). Indeed, the typical reverse-phi response reversal was confined to a limited regime where frequencies were equal and relatively small. We measured 4Q and 2Q detector responses for an identical set of stimuli. The 4Q model predicted positive phi responses and captured response inversion for low-frequency patterns but failed to account for both re-inversion along the diagonal as well as positive responses below the diagonal where flicker exceeds the motion update rate (**Fig 9C**). Importantly, the 2Q detector provided a close quantitative match for our behavioral data without additional tuning. Negative output was restricted to the slow region of the diagonal and the model produced correctly tuned responses below the diagonal (**Fig 9D**). This supports the notion that for a large class of stimuli, a combination of DC level and rectification critically determines response sign and amplitude. Finally, we directly compared behavior and models for stimuli along the diagonal (**Fig 9E**). Response sign and amplitude were generally well explained by the 2Q model but exact temporal tuning differed. Temporal 2Q parameters were initially chosen based on electrophysiological recordings in quiescent flies; due to state modulation, mismatches with data from active animals are not unexpected.

**Fig 10:**
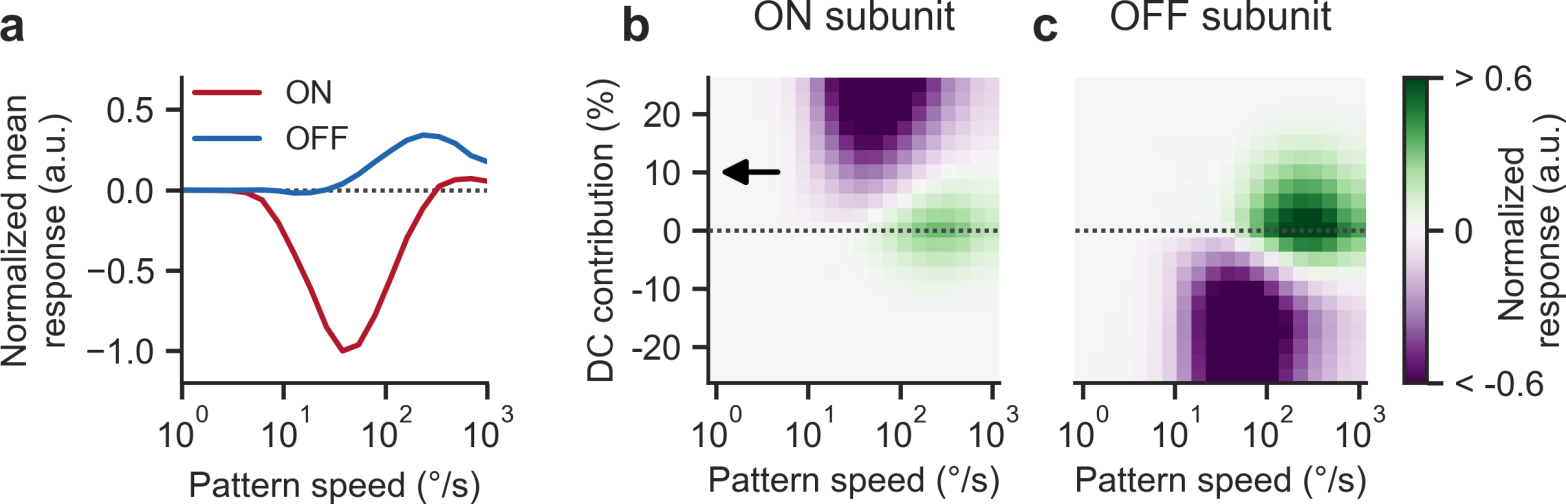
Pathway-specific responses of 2Q model. **a** Velocity tuning curves for reverse-phi pattern (*λ* = 90°) before summation of ON- and OFF-specific channels (ON subunit in red, OFF subunit in blue). Only the ON pathway shows inversion and re-inversion; the OFF pathway consistently indicates physical motion direction. **b** Reverse-phi velocity tuning of the ON subunit as a function of DC contribution. The balance between veridical motion signal and inversion depends on the presence of luminance information at the input; for intermediate levels, positive and negative responses are possible, depending on the particular stimulus velocity. Arrow indicates the default DC parameter of 10%. Values are normalized to a maximum absolute response of one. **c** Equivalent tuning for the OFF subunit. At the standard level of 10%, only positive responses occur. For negative DC, a similar picture as for the ON subunit emerges. Parameters except for DC level are set to their default values. See Materials and Methods for further details on simulations.

In a final set of modeling experiments, we explored the contribution of individual 2Q subunits to the observed reverse-phi responses. Instead of summing ON- and OFF-specific detectors, we determined velocity tuning in isolation (**Fig 10A**). Interestingly, only the ON subunit, biologically corresponding to the difference between oppositely tuned T4 cells, showed reversed and re-inverted responses. The OFF subunit consistently signaled veridical pattern direction, with a tuning curve that resembled the DC-less 2Q detector. Given how tonic sensitivity is implemented in our model, this has a straightforward explanation: after OFF rectification, the invariably positive DC contribution is effectively removed, particularly because we did not implement a rectification offset as in a previous analysis [4]. Only the ON channel then receives information about absolute luminance which we have shown to be critical for reverse-phi sensitivity (**Fig 7C**). If DC is present, non-zero correlations between moving edges and the background occur. The sign of these correlations is, on average, negative [4]. This does not require direct correlation of contrast-switching bars. In addition to these negative signals, bars separated by a time step produce regular phi-type correlations. The balance between the two signals depends, among other factors, on edge density which is determined by the spatial wavelength of the pattern (see **Fig 8**).

If DC is indeed the critical factor in generating reverse-phi responses, then its modulation should strongly affect the behavior of the 2Q detector. We tested this by systematically varying relative DC contribution from strongly positive to strongly negative across stimulus velocities for the reverse-phi condition. The output map generated by the ON subunit exhibited intriguing complexity (**Fig 10B**). As expected, substantial negative DC catastrophically interfered with regular motion responses. If DC was approximately zero, we observed only positive output as we had shown earlier (**Fig 7C**). For positive values at the upper end, responses were uniformly inverted. Interestingly, between these extremes a bilobed transition zone emerged where depending on velocity, output was either negative or positive. This suggests that the biological system operates in a regime where polarity-specific detectors receive an intermediate level of absolute luminance information. Crucially, this picture was approximately inverted when we examined the OFF subunit (**Fig 10C**). The standard DC level of 10% resulted in positive output across the velocity range. By adding a negative DC signal and thus inverted tonic luminance sensitivity, it was possible to smoothly transition toward biphasic and finally purely negative tuning. Overall, we conclude that the biphasic response curve for reverse-phi is determined by the balance between illusory DC-mediated background correlations and veridical phi responses across pattern frames. Stimulus velocity and spatial wavelength jointly influence this balance. DC contribution offers a parameter that continuously regulates the strictness of ON-OFF separation and thus determines the balance between positive and negative output for reverse-phi stimuli.

Taken together, we found that the 2Q detector with DC could fully predict the complex tuning properties of reverse-phi responses in behaving flies. Disabling either rectification or DC component led to an incomplete match with behavioral data.

### T4 and T5 dendrites respond to reverse-phi stimulation

**Fig 11:**
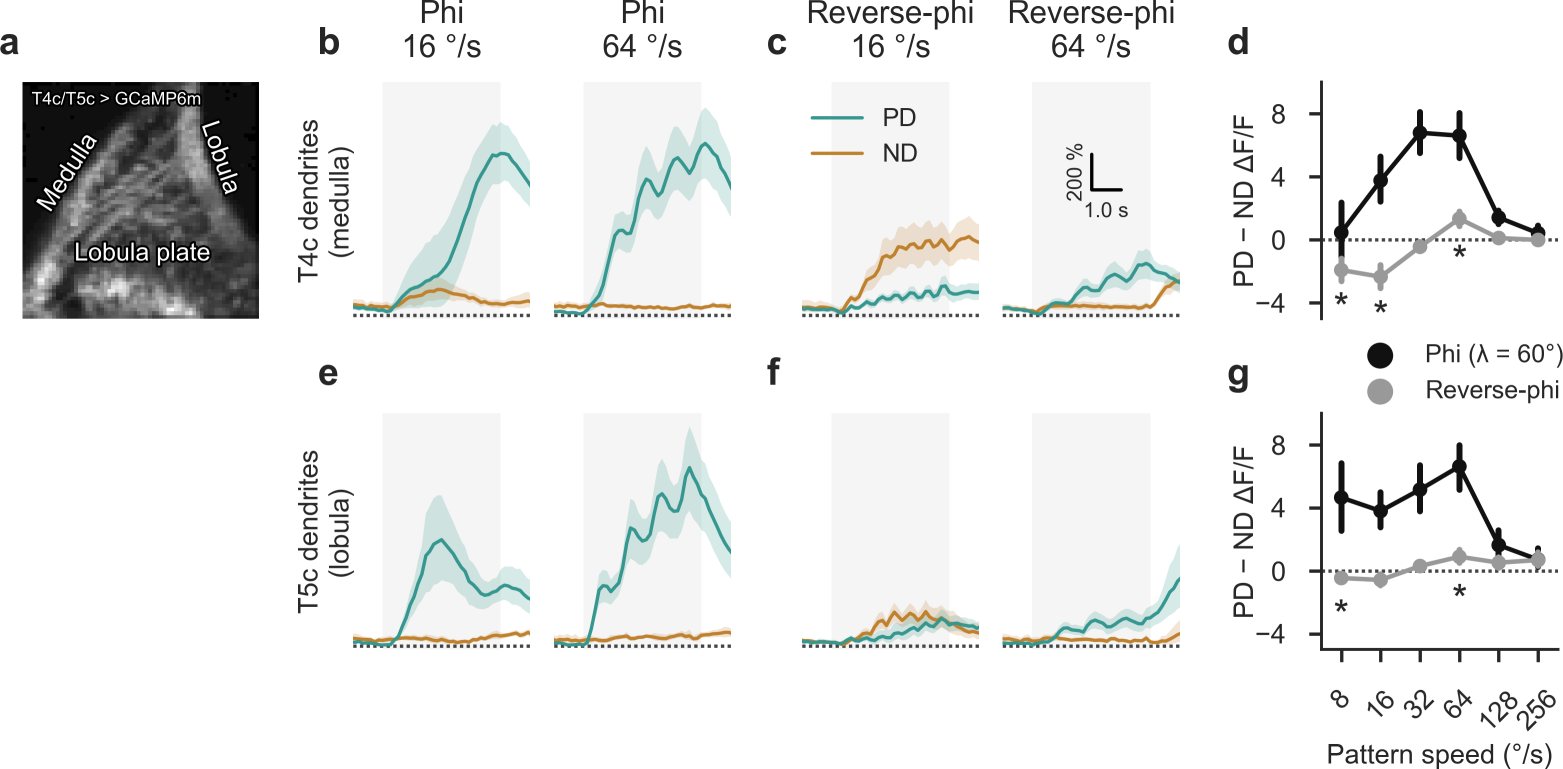
Reverse-phi responses of T4 and T5 dendrites as revealed by *in vivo* calcium imaging. **a** Representative two-photon image of mean GCaMP6m activity within field of view. Only cells targeting the upward-tuned layer 3 of the lobula plate are labeled by the driver line, allowing for isolation of T4 or T5 dendrites through appropriate placement of regions of interest on either medulla or lobula dendrites, respectively. **b** Calcium responses in T4 dendrites (*N* = 9 flies) for phi stimulus at two velocities (*λ* = 60°, step width = 4°). Units are strongly tuned to motion in the preferred (upward) direction. **c** Corresponding T4 responses for reverse-phi motion. Depending on velocity, either PD or ND motion evokes stronger response. **d** Summary of T4 responses, quantified as difference between PD and ND signals (time-averaged between 1 and 4 s after stimulation onset). Calcium activity closely matches behavioral and model results. **e-g** Corresponding results for T5 dendrites. Phi responses resemble those measured in T4. For reverse-phi, a smaller inversion is found. Shaded areas around curves and bars around points indicate 95% confidence intervals. Asterisks show significant differences from zero for reverse-phi responses (Student’s t-test, *P* < 0.05); no tests were performed for phi signals. See Materials and Methods for further details on imaging procedures.

Our standard detector model predicted illusory responses to periodic reverse-phi stimulation only in ON-specific subunits, as our initial parameter choice for DC sensitivity did not lead to tonic responses in OFF input lines. In a subsequent experiment, we traced reverse-phi responses further upstream and imaged *in vivo* calcium activity in T4 and T5 dendrites under a two-photon microscope. A previous study had established reverse-phi sensitivity in these neurons using a random dot motion stimulus [20]. Our interest was focused on potential asymmetries between T4 and T5 as well as the high-velocity re-inversion observed for the periodic stimulus. In order to isolate the two pathways, we relied on a previously established strategy [38,55]: Using GCaMP6m [54] and a driver line that almost exclusively labels T4 and T5 cells of upward-sensitive subtype *c* in layer 3 of the lobula plate, we were able to simultaneously measure direction-selective responses in medulla and lobula dendrites (**Fig 11A**). We presented analogous stimuli as during our behavioral analysis. As expected, responses in T4c dendrites were strongly tuned to motion in their preferred upward direction (**Fig 11B**) in addition to exhibiting velocity tuning (**Fig 11D**). We found pronounced reverse-phi responses for null direction at low and for preferred direction at high velocities which closely matched predictions from behavior and modeling (**Fig 11C and 11D**). These findings confirmed that T4 is already susceptible to the motion illusion. The velocity tuning curve was shifted toward slightly lower pattern velocities, which may be due to state modulation [38,67–69].

OFF-selective T5 dendrites showed similar phi tuning as their T4 counterparts (**Fig 11E–11G**) with comparatively stronger activity at low velocities. However, under reverse-phi stimulation, directional selectivity was substantially smaller. Null direction responses at low pattern speeds were only minimally above those for the preferred direction. At high velocities, in turn, the re-inversion was more pronounced and approached T4 levels. This was in line with the prediction from our original 2Q model which had suggested predominantly ON pathway-mediated reverse-phi responses and primarily veridical output in the OFF pathway.

In summary, we discovered substantial reverse-phi sensitivity and high-velocity re-inversion in T4 dendrites. Corresponding reverse-phi responses in T5, on the other hand, were marginal.

### Pathway-specific blocks affect reverse-phi behavior

**Fig 12:**
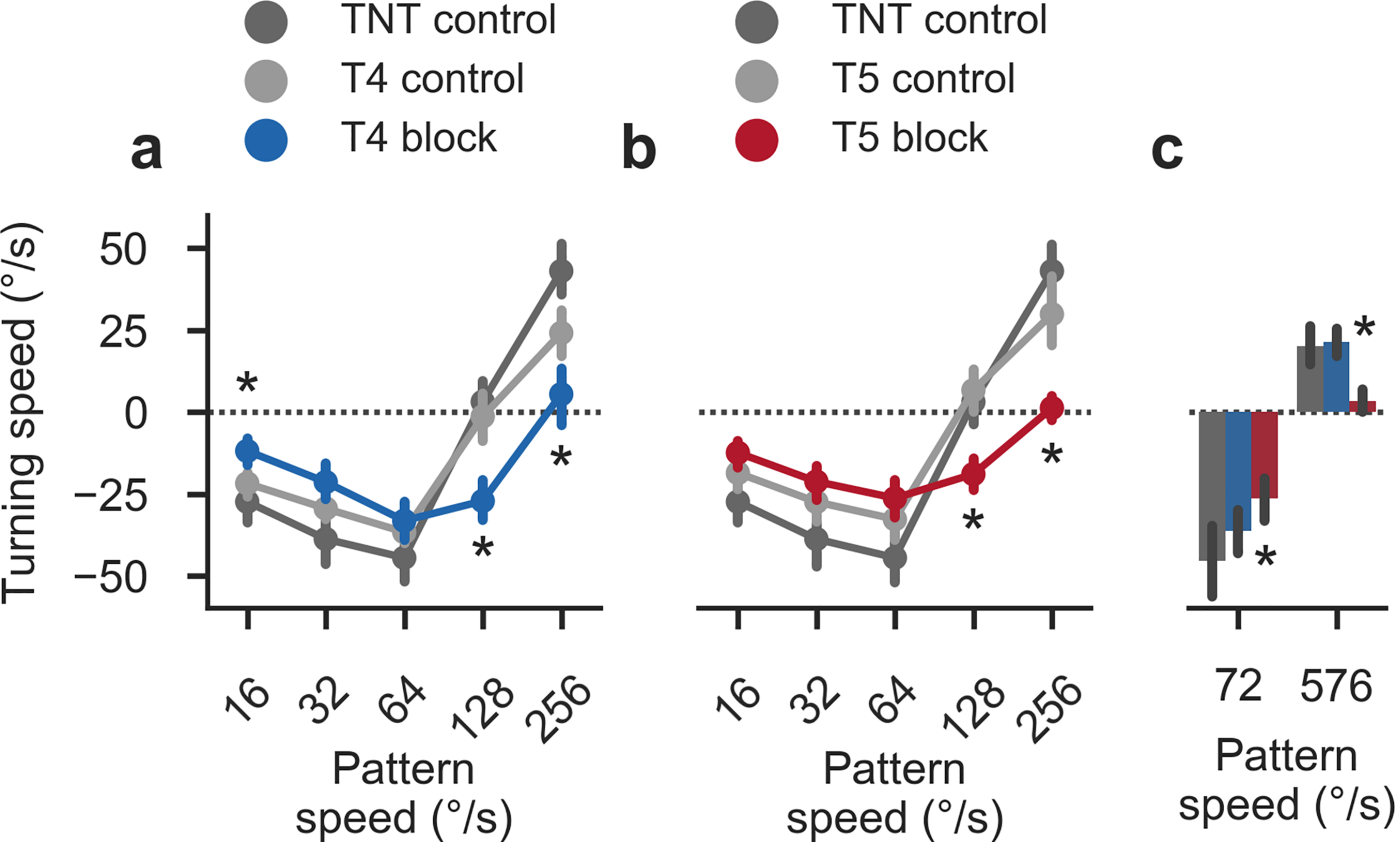
Behavioral responses to reverse-phi stimulation in pathway-specific block flies. **a** Summary statistics for reverse-phi condition (average from 1.5 to 3.0 s after trial onset; *λ* = 90°) in T4 block flies (*N* = 14) and associated controls (*N* = 12/18 for TNT and T4 controls). **b** Summary statistics for reverse-phi condition in T5 block flies (*N* = 15) and associated controls (*N* = 12/12 for TNT and T5 controls). In both T4 and T5 block flies, turning responses are reduced and re-inversion disappears. Bars around points indicate 95% confidence intervals **c** Secondary reverse-phi experiment with increased maximum velocity. For T4 block flies (*N* = 12), high-speed responses are again re-inverted and in line with TNT control flies (*N* = 11). In T5 block flies (*N* = 13), high-speed re-inversion remains abolished. Other stimulus parameters match experiments in **Fig 4**. Lines represent 95% confidence intervals. Asterisks indicate significant differences between T4 or T5 block flies and both appropriate controls (Student’s t-test, *P* < 0.01). See Materials and Methods for further details on behavioral experiments and statistics.

In a final set of experiments, we repeated the initial reverse-phi experiment with flies in which we had silenced either the T4 or T5 pathway. Here, we blocked cells using TNT to stay consistent with earlier work [27,33]. Based on calcium imaging and modeling, we expected a differential effect on reverse-phi sensitivity: with the ON pathway appearing more sensitive to the illusion, T4 silencing should have a disproportionate effect on turning responses when compared to blocking T5. However, both T4 and T5 block flies showed mildly and equally reduced responses (**Fig 12A and 12B**) suggesting either block efficacy insufficient to reveal the effect or indeed interactions not explained by our calcium imaging experiments or the 2Q model. A further complication arises from compressive properties of the sensorimotor transformation [28,66] which could well mask asymmetric silencing effects.

Interestingly, we observed fully abolished responses at the high end of the measured velocity spectrum without the re-inversion found in control flies. Previously, we had established that at the levels of tangential cells as well as behavior, the OFF pathway is tuned to significantly higher velocities than the ON pathway [33]. In order to determine whether re-inverted responses in T4 block flies, whose behavior should be dominated by T5 activity, were simply shifted outside our measured velocity range, we ran a second set of experiments in which we showed both an intermediate and the maximum attainable reverse-phi velocity (**Fig 12C**). At the most extreme pattern speed, T4 block flies in which mostly T5 should contribute to optomotor responses did show turning responses while T5 block fly behavior remained reduced. This was in line with expectations from channel asymmetries. Intriguingly, the finding also suggests that the contribution of T5 to the re-inversion outweighs that of T4, possibly reflecting stronger veridical motion components in the OFF pathway (**Fig 10A**).

## Discussion

The reverse-phi illusion offers a deep look into computational details of motion detection. Here, we made an effort to bridge the extant gap between algorithm and neural circuit further. First, in a set of behavioral experiments, we generalized results from flight to overall locomotion by showing that walking *Drosophila* robustly respond to reverse-phi stimulation. Critically, turning tended to re-invert for high update frequencies. Silencing of local ON and OFF motion detector cells T4 and T5 fully abolished phi as well as reverse-phi responses. When we recorded from global flow field detectors in the lobula plate, primary recipients of T4 and T5 inputs, we found them to respond to reverse-phi stimulation. We tested the ability of Reichardt-type elaborated motion detection models to account for general and detailed aspects of reverse-phi behavior. A rectified two-quadrant detector with DC contribution at the input stage reproduced both contrast-induced reversal and high-frequency re-inversion. Removing either DC or rectification abolished aspects of the response, suggesting that a combination of the two architectural features gives rise to the full spectrum of reverse-phi responses. This confirms and extends our previous work on apparent motion [4]. Furthermore, we were able to show that calcium activity in T4 and, to a lesser extent, T5 dendrites reflects the bilobed shape of the reverse-phi velocity tuning curve, thus accounting for such sensitivity at subsequent levels. In summary, we propose that ON- and OFF-channel output units T4 and T5 mediate reverse-phi sensitivity and that a simple two-quadrant model is sufficient to explain ON-OFF interactions for a wide range of such stimuli.

Our model analysis revealed moderate DC input as the key component for explaining both response reversal as well as high-frequency re-inversion. Indeed, the combination of DC and subsequent half-wave rectification represents a strong non-linearity capable of altering response properties drastically in certain stimulus regimes (see, for instance, **Fig 10B and 10C**). We currently lack a comprehensive understanding of how such tonic components are generated and propagated through the circuits presynaptic to T4 and T5. However, for a subset of T4 and T5 input cells, there is compelling evidence of sensitivity to absolute light level. Electrophysiological recordings from medulla interneurons Mi1 and Tm3, providing critical input to T4 [35,37], indicate preserved DC components whose magnitude is on the order of 10–20% relative to the peak of transient responses [32]. This matches well with parameters used here and in previous work [4]. Moreover, calcium imaging from a comprehensive set of medulla cells presynaptic to T4 and T5 reveals Mi4, Mi9, and Tm9 to be purely DC-sensitive and without discernible high-pass characteristic [28,38,70]. Interestingly, in T5 reverse-phi responses were subdued compared to T4. The suggested model can account for differential reverse-phi sensitivity by assuming limited contribution of DC in the OFF pathway. An investigation of lamina cell involvement in various visual behaviors shows that genetic manipulation of feedback cells C2 and C3 affects the re-inversion at high velocities observed here and previously [6,34]. Such feedback cells may be involved in titrating tonic luminance sensitivity, thereby shaping the tuning of reverse-phi responses. Overall, we consider persistence of DC up to the non-linear stages of motion detection plausible. The issue that such leaky rectification is at odds with the precisely polarity-specific edge responses observed in T4 and T5 [20,27,33,71] remains unresolved.

Previous studies have put forward alternative models to account for reverse-phi responses in behaving fruit flies. We see a critical advantage in the straightforward architectural plausibility of our approach: each subunit of the rectified 2Q model finds direct correspondence in either the T4 or the T5 pathway. 4Q-type models [5,6] either assume sign-correct multiplication, which appears biophysically prohibitive, or include equally weighted subunits that compute non-linear interactions between ON and OFF. For this, neural correlates have not been discovered. Moreover, data from T4/T5 block flies in which optomotor behavior is selectively and fully abolished suggest that at the circuit level, no such cross-over interactions are represented [33,56,57]. In our model, ON-OFF interaction arises from limited cross-polarity sensitivity conferred by DC contributions. Critically, such models can account for the subtle effect of high-frequency re-inversion without modification.

Previous work substituted a true time delay for linear filtering to recover the effect in a 4Q model [6]. This type of temporal filtering appears difficult to realize in any biological circuit and physiological recordings from relevant input neurons indicate smooth impulse responses [28,32,38]. Additionally, models that involve pure phase shifts are subject to strong temporal aliasing which is not observed in either optomotor responses or recordings from tangential cells [72]. Our examination of filter settings did not yield re-inverted responses for the regular 4Q architecture (**Fig 7F–7I**). However, these findings do not prove the impossibility of such a detector. Indeed, we cannot rule out other plausible algorithmic modifications that explain the biphasic response curve: the space of potential elaborated detectors is, after all, large. Here, we offer a simple, neurally grounded model that can account for a majority of response properties. Finally, our detector does not incorporate various properties of the fly visual system, such as luminance adaptation, non-linear spatial integration of motion detectors, or the emerging multitude of differentially tuned T4/T5 inputs [1,28,32,38]. Future models will have to take this complexity into consideration.

We have recently offered evidence that the tuning of ON and OFF motion pathways differs and that these disparities may represent adaptation to the statistics of natural surrounds [33]. Here, to keep the model parsimonious as well as to allow direct comparison with earlier work on apparent motion, we retain polarity-symmetric parameters [4]. Given that our asymmetric detector includes a DC component of similar strength as well as largely similar filter parameters, we predict our findings should carry over. Interestingly, the optimization procedure yielded negative DC values for the OFF pathway. Reversed sensitivity to tonic luminance not removed by rectification would explain the small reverse-phi responses we observed in T5 dendrites. Furthermore, we have shown before that T4/T5 block flies do not respond to so-called two-point glider stimuli of negative parity. These patterns are one possible instantiation of the reverse-phi principle; that is, they are equivalent to random binary first-order motion with regular contrast reversals [33,58,73]. Additionally, a recent study demonstrated that behavioral responses to random dot reverse-phi stimuli are abolished in T4/T5-silenced flies [20]. The stimuli used here, however, allowed us to pursue another constraint: high-velocity re-inversion.

Earlier studies have put forward that reverse-phi sensitivity may represent a solution for noise suppression. In elementary motion detection schemes, the non-linearity tends to enhance spurious input fluctuations [6,74]. It is possible that suitable DC components provide an approximation of such explicit ON-OFF interactions, thereby improving coding of motion signals corrupted by photon or neural noise.

Most recently, high-precision stimulation of individual neuro-ommatidia as well as system identification techniques have shown the non-linearity that generates direction selectivity on T4 or T5 dendrites to be more elaborate than anticipated [55,75]. Specifically, the two cells make use of both preferred direction enhancement and null direction suppression to shape responses. Our model only captures the former by means of a multiplicative non-linearity. However, for many periodic stimuli the two models behave rather similarly. It seems likely that a more elaborate combined motion detector would produce similar output as our 2Q model does under periodic reverse-phi stimulation, assuming equivalent processing of inputs.

Finally, reverse-phi effects are found in many organisms and on levels ranging from basic visual responses and reflexes up to conscious perception. Motion detection in the mammalian retina is thought to rest on similar computational principles as analogous circuits in the fly sensory periphery [2,76]. In particular, a similar circuit division between ON motion- and OFF motion-processing units appears to be at play. Various mechanisms have been suggested to account for reverse-phi sensitivity, particularly in the human visual system. It will be interesting to explore whether appropriately designed gradient-based schemes, for instance, are capable of explaining the particular effects observed here [6,21–24]. However, in light of strong evidence for correlation-based motion detection in the fly [1,77], our present work focuses on the suitably elaborated Hassenstein-Reichardt detector. This algorithm is closely related and, under mild assumptions, mathematically equivalent to a successful class of models for *de novo* direction selectivity in visual cortex based on the notion of motion energy [14,39]. The components that give rise to reverse-phi sensitivity in our model are biophysically simple. We think that similar processing may illuminate aspects of mammalian and specifically human reverse-phi sensitivity. However, whether this basic circuit model does indeed generalize to high-level representations of motion awaits investigation.
